# Maltol Improves Peripheral Nerve Function by Inhibiting Schwann Cell Apoptosis via the PERK/eIF2α/CHOP Pathway and MME Upregulation in Diabetic Peripheral Neuropathy

**DOI:** 10.3390/ph17091139

**Published:** 2024-08-29

**Authors:** Jiawei Li, Quan Liu, Shuainan Liu, Hong Xin, Xuemei Zhang, Nan Guo

**Affiliations:** 1 Minhang Hospital, School of Pharmacy, Fudan University, Shanghai 201203, China; 22211030070@m.fudan.edu.cn (J.L.); xinhong@fudan.edu.cn (H.X.); 2 Key Laboratory of Polymorphic Drugs of Beijing, Institute of Materia Medica, Chinese Academy of Medical Sciences and Peking Union Medical College, Beijing 100050, China; popliu@imm.ac.cn (Q.L.); liusn@imm.ac.cn (S.L.)

**Keywords:** maltol, apoptosis, MME, ER stress, Schwann cells, diabetic peripheral neuropathy

## Abstract

Diabetic peripheral neuropathy (DPN) is the most prevalent chronic complication among diabetic patients and a primary risk factor contributing to the deterioration of diabetic foot conditions. The pathogenesis of DPN remains complex and not fully understood, and there are hardly any effective treatment drugs. Maltol (3-hydroxy-2-methyl-4-pyranone) has demonstrated antioxidant and anti-inflammatory properties. However, the potential role of maltol in the treatment of DPN remains unclear. This study aimed to assess maltol’s effects on DPN rats and high glucose (HG)/palmitic acid (PA)-induced rat Schwann cells (RSC96). The results indicated maltol’s capacity to enhance peripheral nerve function in DPN rats. In RSC96 cells stimulated with high HG and PA, maltol treatment reduced DPN markers and apoptosis-related proteins. Functional enrichment analysis of differentially expressed genes revealed that endoplasmic reticulum (ER) stress pathways were involved in this process. Western blot results demonstrated the activation of ER stress pathway in HG/PA-induced RSC96 cells, with maltol attenuating ER stress-related protein expression. Furthermore, the knockdown of Membrane metallo-endopeptidase (MME) reversed maltol’s effects on apoptosis-related protein expression, suggesting a potential therapeutic role for maltol via MME in treating DPN. These findings indicate that maltol may hold promise as a therapeutic agent for DPN treatment.

## 1. Introduction

Diabetic peripheral neuropathy (DPN) is a prevalent chronic complication of diabetes [1], characterized by symptoms such as neuropathic pain, paresthesia, tingling, numbness, and burning sensations [2]. These symptoms primarily affect the toes, feet, or legs, significantly impacting morbidity, mortality, and the quality of life [3]. Despite its high incidence, the pathophysiological mechanisms of DPN have not been fully elucidated. There are currently no approved treatments or drugs specifically targeting DPN.

It is reported that the pathogenesis of DPN includes metabolic disorders, oxidative stress, inflammation, vascular dysfunction, and the accumulation of advanced glycation end products (AGEs), which are all closely related to the development of DPN [4,5,6,7,8]. Recent evidence has highlighted endoplasmic reticulum (ER) stress as a significant molecular mechanism in the pathogenesis of DPN [9]. The ER is responsible for the correct folding of polypeptide chains and the processing of secretory proteins. Various exogenous or endogenous factors such as elevated blood glucose and hypoxia can lead to the accumulation of misfolded or unfolded proteins in the ER, resulting in ER stress [10]. The unfolded protein response (UPR) is activated following ER stress, and the three primary sensors that regulate UPR include activated transcription factor 6 (ATF6), protein kinase RNA-like ER kinase (PERK), and inositol-requiring enzyme 1 (IRE1) [11]. The ER-resident chaperone glucose-regulated protein 78 (GRP78), also known as binding immunoglobulin protein (BiP), typically binds to the ER luminal domain of these sensors, keeping them inactive [11,12]. Under conditions of misfolded protein accumulation, GRP78 dissociates, leading to the activation of ATF6 and PERK, which phosphorylate eukaryotic translation initiation factor 2 subunit 1 (eIF2α), and finally the activation of the transcription factor C/EBP homologous protein (CHOP) [12]. The activation of CHOP protein leads to the activation of proapoptotic genes and to cell cycle arrest, DNA damage, and apoptosis [11].

Maltol (3-hydroxy-2-methyl-4-pyranone) is an aromatic compound widely distributed in nature, with particularly high levels found in red ginseng. It is extensively utilized as a safe flavoring agent and food preservative. Maltol exhibits diverse pharmacological effects, including protection against cisplatin-induced nephrotoxicity by reducing oxidative stress [13]. Additionally, maltol has demonstrated therapeutic potential in conditions such as osteoarthritis, liver fibrosis, and the aging of the liver and kidneys [14,15,16]. In terms of neuroprotection, maltol acts through pathways involving NF-κB and mitogen-activated protein kinase signaling [17,18].

Membrane metallo-endopeptidase (MME) is a 100 kDa type II transmembrane protein containing a conserved zinc-binding motif in its extracellular C-terminal domain, which is involved in degrading various substrates [19,20]. MME is widely expressed in normal tissues, particularly in peripheral nerves [21]. Studies, such as Cai’s research, suggest that the upregulation of MME can mitigate kidney inflammation and apoptosis induced by high glucose, revealing that MME may be a potential molecule in diabetes [22]. MME knockout mice show increased amyloid beta peptides, suggesting a potential role in Alzheimer’s disease prevention [23]. These results indicate that MME is involved in various biological processes such as inflammatory response and neurodevelopment.

Our study aimed to elucidate whether maltol can offer protection against DPN and investigate potential mechanisms both in vivo and in vitro. The current research demonstrated the role of maltol on DPN, and Gene Ontology (GO) analysis suggested that the process may be mediated by relieving ER stress, which we verified in vitro. Meanwhile, the analysis of differential genes by gene chip showed that maltol may alleviate DPN by increasing the expression of MME.

## 2. Results

### 2.1. Maltol Improved Peripheral Nerve Function in Diabetic Rats

**Figure 1:**
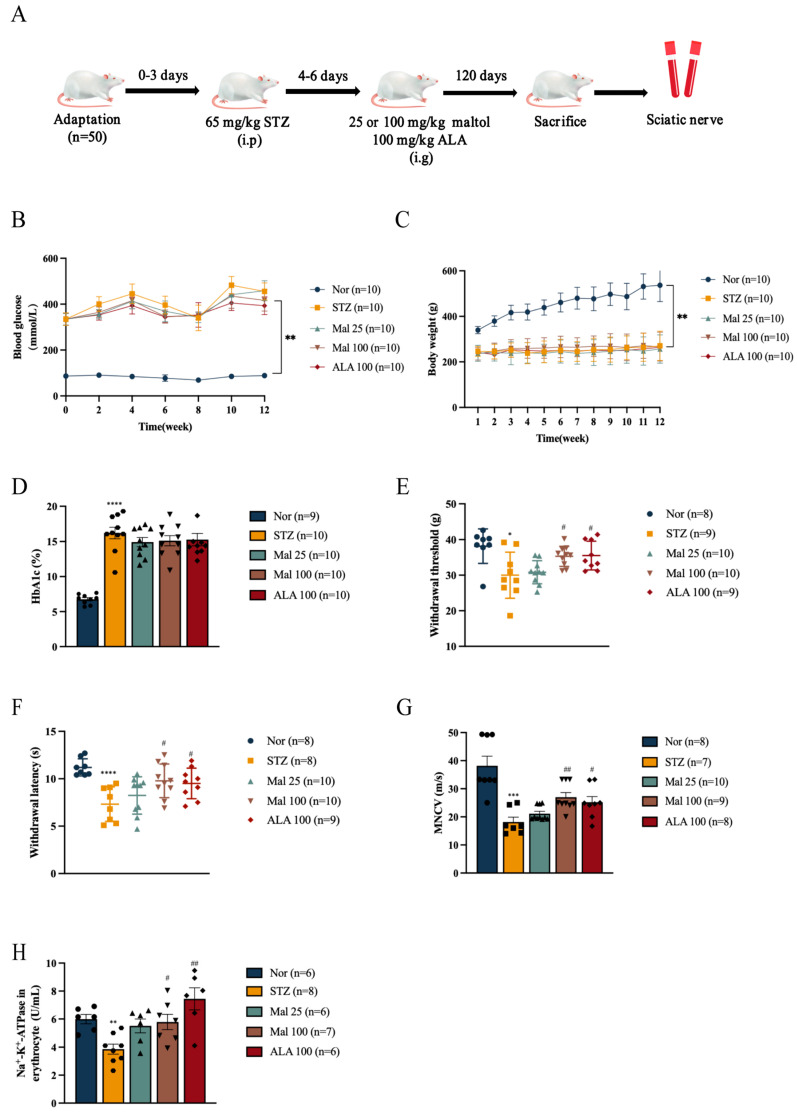
Maltol improved peripheral nerve function in diabetic rats. (**A**) Administration of maltol in diabetic rats. (**B**,**C**) Body weight (**B**) and blood glucose (**C**) in rats. (**D**) HbA1c levels in rats. (**E**) Mechanical threshold detection in rats. (**F**) Thermal threshold detection in rats. (**G**) Motor nerve conduction velocity (MNCV) detection in sciatic nerves of rats. (**H**) Na^+^-K^+^-ATPase activity in rats. Mal: maltol. ALA: α-Lipoic acid. Symbols of each group represent different rats. Data are expressed as mean ± SEM, *n* = 10, * *p* < 0.05, ** *p* < 0.01, *** *p* < 0.001, **** *p* < 0.0001 compared with Nor group; # *p* < 0.05, ## *p* < 0.01 compared with STZ group.

The DPN model was induced via a Streptozocin (STZ) injection (Figure 1A). Blood glucose levels were markedly elevated in the STZ-induced diabetic rats compared to the normal rats (Figure 1C). Following the maltol administration to the diabetic rats, no significant differences were observed in body weights and glucose levels between the STZ and Mal groups (Figure 1B,C). Similarly, the measurement of HbA1c levels showed no significant difference between the STZ and Mal groups (Figure 1D). Compared to the normal rats, the STZ-induced diabetic rats exhibited decreased withdrawal threshold and latency in response to mechanical and thermal stimuli (Figure 1E,F). In contrast, the repeated administration of maltol at doses of 25 mg/kg and 100 mg/kg over a 12-week period significantly increased withdrawal threshold and latency in response to mechanical stimulation and thermal stimulation, suggesting the attenuation of pain hyperalgesia compared to the STZ-induced diabetic rats (Figure 1E,F). Furthermore, motor nerve conduction velocity (MNCV) in the sciatic nerves of the diabetic rats increased by 1.48-fold following the administration of 100 mg/kg maltol (Figure 1G). Na^+^-K^+^-ATPase activity, which serves as an indicator of red blood cell deformability and peripheral nerve injury and is a potential biomarker for DPN [24], was significantly enhanced by maltol treatment, indicating the amelioration of STZ-induced peripheral nerve injury (Figure 1H).

### 2.2. Maltol Alleviates STZ-Induced Oxidative Stress in Rats

**Figure 2:**
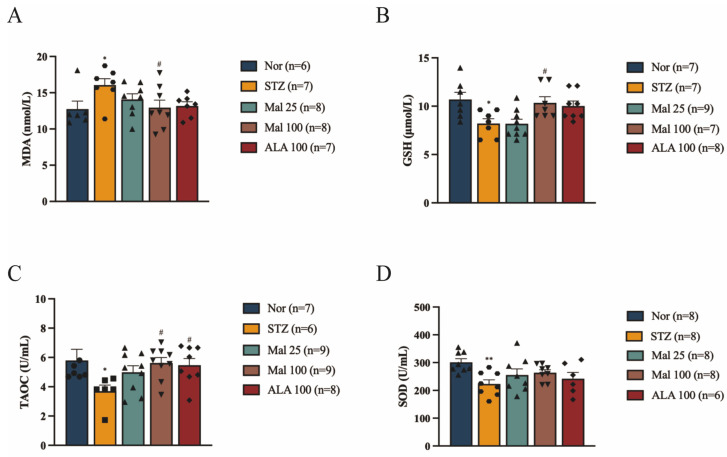
Maltol alleviates STZ-induced oxidative stress in rats. (**A**) MDA levels in rats. (**B**) GSH levels in rats. (**C**) TAOC levels in rats. (**D**) SOD levels in rats. Mal: maltol. ALA: α-Lipoic acid. Symbols of each group represent different rats. Data are expressed as mean ± SEM, *n* = 10, * *p* < 0.05, ** *p* < 0.01 compared with Nor group; # *p* < 0.05 compared with STZ group.

Compared to the normal rats, the STZ-induced diabetic rats exhibited significant oxidative and antioxidant imbalances, characterized by increased serum MDA levels and decreased serum TAOC, GSH, and SOD levels (Figure 2A–D). Following 12 weeks of maltol administration, there was a significant dose-dependent increase in serum TAOC, GSH, and SOD levels, along with a decrease in serum MDA levels compared to the STZ-induced diabetic rats (Figure 2A–D). These findings suggest that maltol may treat DPN by protecting against STZ-induced oxidative damage.

### 2.3. Protective Effect of Maltol on the Viability of HG/PA-Exposed RSC96 Cells

**Figure 3:**
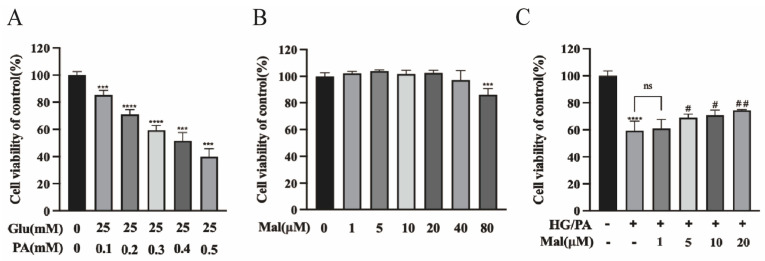
Protective effect of maltol on the viability of HG/PA-exposed RSC96 cells. (**A**) The viability of HG/PA-exposed RSC96 cells with CCK-8 assay. (**B**) The viability of maltol-exposed RSC96 cells at various concentrations for 24 h using a CCK-8 assay. (**C**) The cell viability after treatment with 25 mM glucose and 0.3 PA, with maltol at different concentrations, using a CCK-8 assay. Glu: glucose; PA: palmitic acid; HG: high glucose; Mal: maltol. Data are expressed as mean ± SEM, *** *p* < 0.001, **** *p* < 0.0001 compared with Nor group; # *p* < 0.05, ## *p* < 0.01 compared with HG/PA group.

To evaluate the protective effect of maltol on the viability of RSC96 cells exposed to HG and PA, cells were incubated with increasing concentrations of PA (0.3 mM) and 25 mM glucose for 24 h, followed by an assessment of cell viability using the CCK-8 assay. As shown in Figure 3A, cell viability decreased to approximately 60% under these conditions, which was used as the subsequent model concentration. To assess the potential cytotoxicity of maltol in cultured cells, RSC96 cells were treated with varying concentrations (1, 5, 10, 20, 40, and 80 µM) of maltol for 24 h, and cell viability was measured using the CCK-8 assay. The results indicated no significant cytotoxicity at concentrations ranging from 1 to 40 µM (Figure 3B). Furthermore, maltol significantly improved RSC96 cell viability after 24 h of incubation with HG and PA, confirming its protective effect against HG/PA-induced cytotoxicity (Figure 3C).

### 2.4. Maltol Increased Nerve Growth Factor and Neuritin-1 Expression and Inhibited Apoptosis in HG/PA-Induced RSC96 Cells

**Figure 4:**
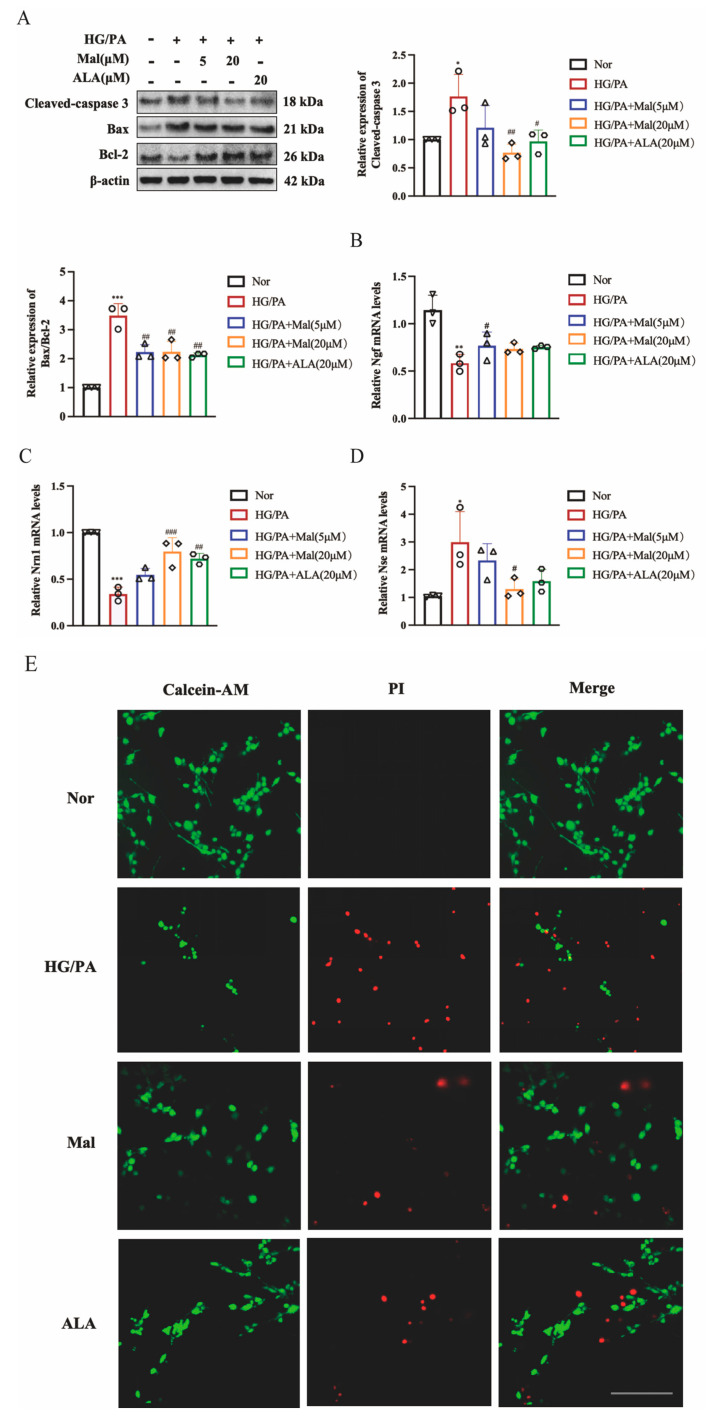
Maltol increased nerve growth factor and neuritin-1 expression and inhibited apoptosis in HG/PA-induced RSC96 cells. (**A**) Western blot detection of Bax, Bcl-2, and Cleaved caspase-3 in RSC96 cells. (B,C) Real-time PCR detection of ngf (**B**) and nrn1 (**C**) mRNA expression in RSC96 cells. (**D**) Real-time PCR detection of NSE mRNA expression in RSC96 cells. (**E**) Evaluation of cell viability using the Calcein-AM/PI Double Stain Kit to distinguish live cells (green) from dead cells (red). Scale bar = 100 μm. PA: palmitic acid; HG: high glucose; Mal: maltol. ALA:α-Lipoic acid. Symbols of each group represent the number of repetitions of the experiment. Data are expressed as mean ± SEM, * *p* < 0.05, ** *p* < 0.01, *** *p* < 0.001 compared with Nor group; # *p* < 0.05, ## *p* < 0.01, ### *p* < 0.001 compared with HG/PA group.

Nerve growth factor (NGF) is the prototypical growth factor within the neuronutrient cohort, essential for the maturation and maintenance of cholinergic neurons in both the peripheral and central nervous systems [25]. NGF has been documented to alleviate neuropathic symptoms by protecting peripheral nervous system neurons and restoring their function [26]. Neurotrophin 1 (Nrn1) is crucial in neurodevelopment and regeneration, promoting neuron proliferation and synaptic refinement [27]. Schwann cells possess the capability to secrete NGF and Nrn1, enhancing the survival and regeneration of neurons. We investigated the protective effect of maltol on neuromodulators in peripheral neuropathy and observed that maltol significantly enhanced the downregulation of NGF and Nrn1 caused by HG and PA (Figure 4B,C). Additionally, levels of neuron-specific enolase (Nse), a biomarker for nerve injury involved in the conversion of 2-phosphoglyceric acid to phosphoenolpyruvate, and an enzyme specific to neurons and neuroendocrine cells were assessed across experimental groups [28]. A prolonged hyperglycemic environment can lead to the neuronal damage and immediate release of NSE. Therefore, elevated NSE levels may represent the occurrence of DPN disease [28]. Maltol supplementation reversed the diabetes-induced upregulation of NSE production, as shown in Figure 4D. Bcl-2-associated X protein (BAX) and B-cell lymphoma-2 (Bcl-2) belong to the Bcl-2 family and regulate mitochondrial membrane permeability to determine whether to initiate apoptosis [29]. Caspases, a family of cysteine proteases, are key mediators of programmed cell death or apoptosis, and Cleaved Caspase-3 is the activated form of Caspase-3 [30]. Subsequent analysis of apoptosis-related proteins revealed a significant increase in Cleaved caspase-3 and Bax expression, accompanied by a decrease in Bcl-2 levels in RSC96 cells exposed to HG and PA (Figure 4A). Interestingly, maltol treatment mitigated these effects in Figure 4A. The results from the Calcein-AM/PI Double Stain further confirmed that maltol reduced apoptosis in RSC96 cells (Figure 4E).

### 2.5. Functional Annotation of Differentially Expressed Genes in Sciatic Nerve

**Figure 5:**
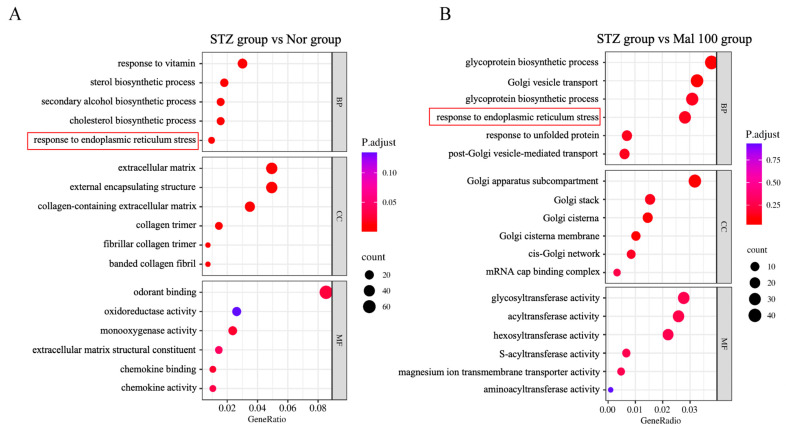
Functional annotation of differentially expressed genes in sciatic nerve. (**A**) GO analysis for functional annotation of differential genes in STZ group versus Nor control group. (**B**) GO analysis for functional annotation of differential genes in STZ group versus Mal 100 group. The red boxs represent consistent enrichment pathways. Data are expressed as mean ± SEM, *n* = 1.

To elucidate the functional pathways associated with differential gene expression, we conducted GO analysis for a functional annotation of genes in the STZ versus normal control group and the STZ versus maltol administration group. GO analysis categorizes genes into three branches: Biological Process, Cellular Component, and Molecular Function. The number of differential genes in each GO category was counted, and enrichment analysis was performed to assess the significance of differential gene enrichment. As depicted in Figure 5A,B, both STZ versus normal and STZ versus maltol administration groups showed consistent enrichment in endoplasmic reticulum (ER) stress-related pathways. These findings suggest that maltol may exert an anti-apoptotic effect by modulating ER stress pathways.

### 2.6. Maltol Treatment Attenuated HG/PA-Induced Excessive ER Stress

**Figure 6:**
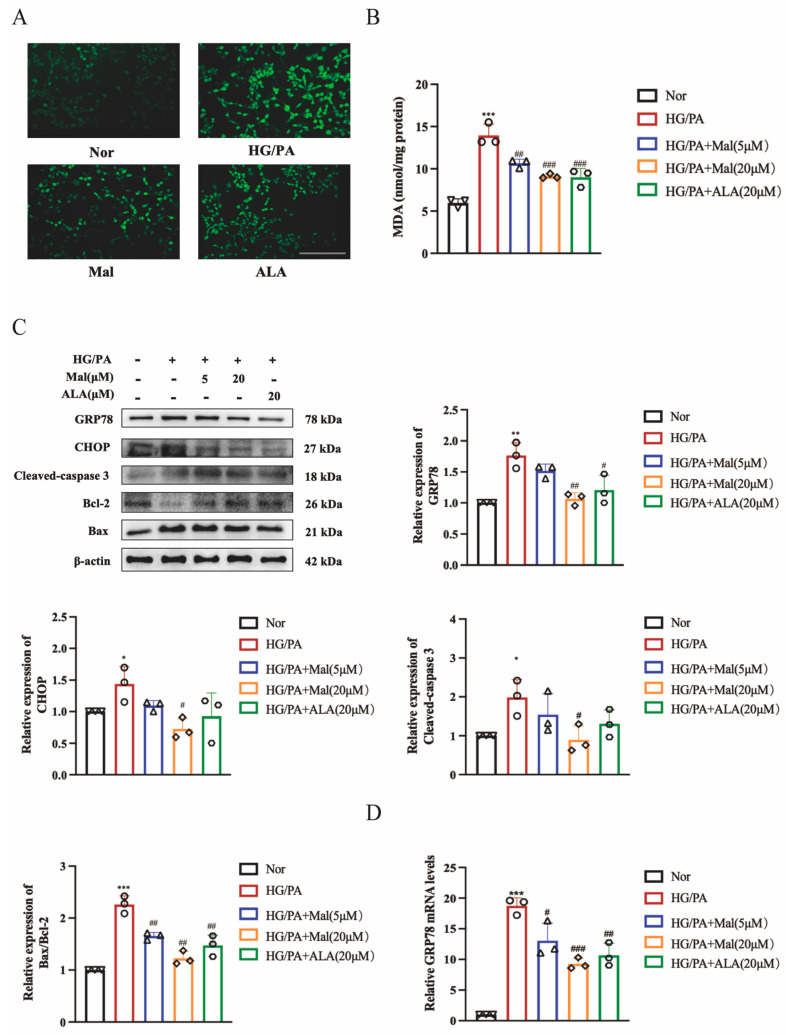

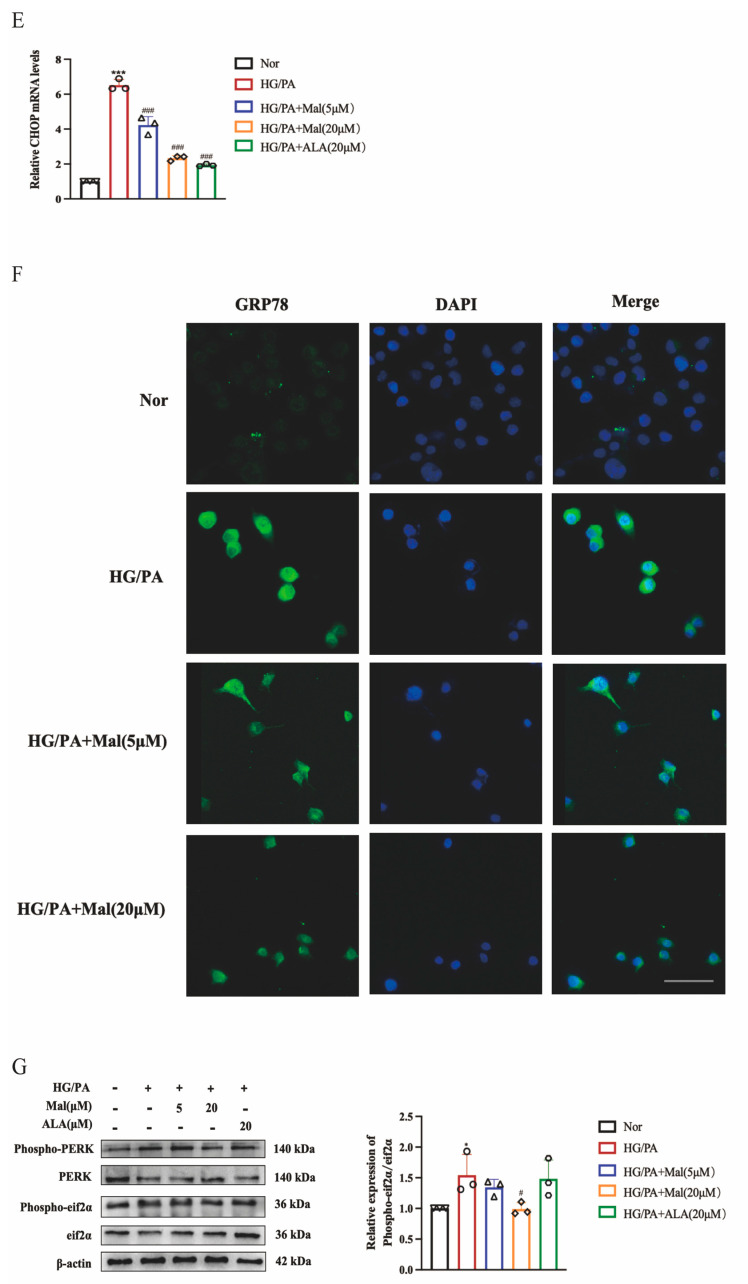

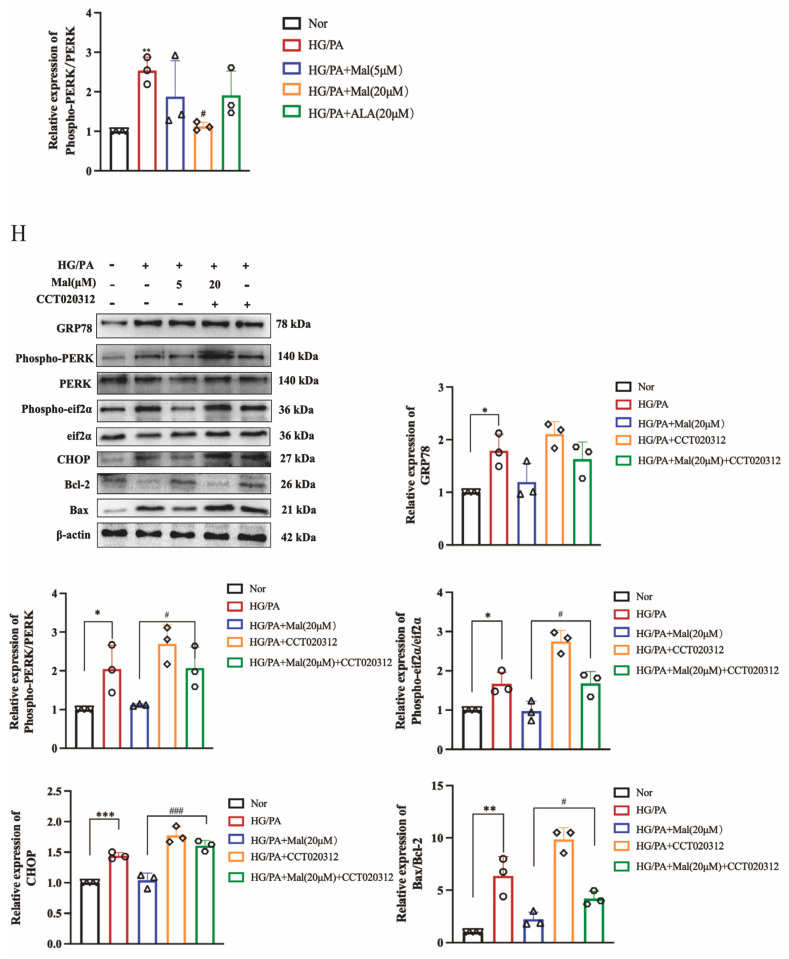
Maltol treatment attenuated HG/PA-induced excessive ER stress. (**A**) Detection of ROS (green) production by the ROS Assay Kit in RSC96 cells. (**B**) MDA levels of RSC96 cells. (**C**) Western blot detection of GRP78, CHOP, Bax, Bcl-2, and Cleaved caspase-3 in RSC96 cells. (**D**,**E**) Real-time PCR detection of GRP78 (**D**) and CHOP (**E**) mRNA expression in RSC96 cells. (**F**) Immunofluorescence of GRP78 expression in RSC96 cells. (**G**) Western blot detection of Phospho-eIF2α, eIF2α, Phospho-PERK, and PERK in RSC96 cells. (**H**) Western blot detection of Phospho-eIF2α, eIF2α, Phospho-PERK, and PERK in RSC96 cells. Scale bar = 100 μm. PA: palmitic acid; HG: high glucose; Mal: maltol. ALA:α-Lipoic acid. Symbols for each group represent the number of repetitions of the experiment. Data are expressed as mean ± SEM, * *p* < 0.05, ** *p* < 0.01, *** *p* < 0.001 compared with Nor group; # *p* < 0.05, ## *p* < 0.01, ### *p* < 0.001 compared with HG/PA group.

Excessive production of reactive oxygen species (ROS) can disrupt the REDOX balance and promote oxidative damage in DPN [31]. Here, we employed 2′,7′-dichlorodihydrofluorescein diacetate (DCFH-DA) to assess ROS formation, where ROS oxidizes DCFH-DA into fluorescent DCF. Our results demonstrated that maltol treatment significantly decreased ROS formation in RSC96 cells stimulated with HG and PA (Figure 6A). To measure lipid peroxidation, we used an MDA assay kit and found that high glucose conditions increased MDA levels in RSC96 cells. However, maltol administration significantly reduced MDA content compared to the HG/PA group, indicating its potential to ameliorate oxidative stress associated with diabetes (Figure 6B). Oxidative stress and ER stress are interconnected processes, with ER stress emerging as a novel mechanism in the initiation and progression of DPN [9]. To validate our GO analysis results, we examined the protein levels of the ER stress markers GRP78 and the ER stress-related apoptosis protein CHOP. Our study revealed that maltol reduced the expression of GRP78 and CHOP. Furthermore, maltol increased the expression of Bcl-2 and decreased the expression of Bax, indicating its role in mitigating apoptosis (Figure 6C). Similarly, maltol also reduced the expression of Cleaved caspase-3 (Figure 6C). We also assessed GRP78 and CHOP expression in RSC96 cells exposed to HG/PA using RT-qPCR. Intracellular levels of GRP78 and CHOP were significantly elevated in the HG/PA group compared to the Nor group but significantly reduced in the HG/PA+Maltol group compared to the HG/PA group (Figure 6D,E). The immunofluorescence results were consistent with these findings (Figure 6F). Given that maltol decreased HG-induced ER stress-related apoptotic protein CHOP, we hypothesized whether this effect was mediated by downregulating upstream proteins. PERK is one of the UPR transmembrane proteins that induces CHOP expression by promoting eIF2α expression. Once GRP78 is released, PERK begins oligomerization activation within the ER membrane, inducing its autophosphorylation and activating the kinase domain [12]. Our results showed that maltol attenuated the phosphorylation of PERK and eIF2α (Figure 6G), suggesting that maltol inhibits ER stress-induced apoptosis by downregulating PERK and eIF2α expression. Further investigating the mechanism of maltol action in HG/PA-induced RSC96 cells, we used the PERK activator CCT020312. Interestingly, while PERK expression remained unchanged, the levels of phospho-PERK, CHOP, GRP78, and Bax were significantly increased, and Bcl-2 expression was decreased in the HG/PA+Maltol+CCTO20312 group compared to the HG/PA+Maltol group (Figure 6H). These results indicate that maltol exerts its anti-apoptotic effects through the modulation of the endoplasmic reticulum stress pathway.

### 2.7. Bioinformatics Analysis of Potential Maltol Drug Target Associated with DPN

**Figure 7:**
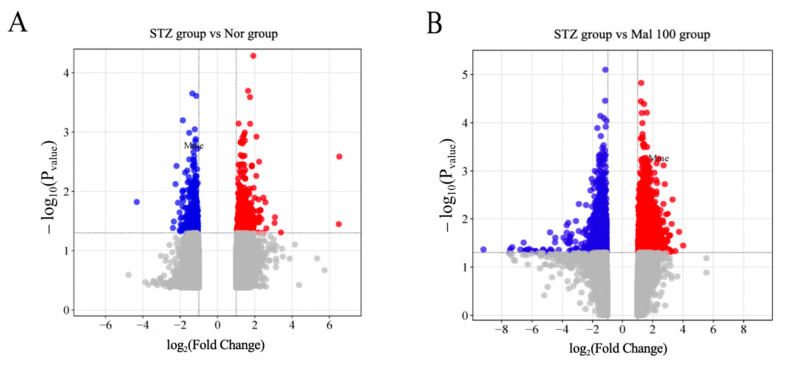
Bioinformatics analysis of potential maltol drug target associated with DPN. (**A**) Differentially expressed genes (DEGs) in STZ group versus Nor control group. (**B**) Differentially expressed genes (DEGs) in STZ group versus Mal 100 group. The red dots are upregulated differential genes, and the blue dots are downregulated differential genes. Data were expressed as mean ± SEM, *n* = 1.

Gene chip analysis was employed to identify differential genes among the maltol administration group, STZ group, and normal control group, using a screening criterion of *p*-value < 0.05. In the STZ versus normal control group, we identified 849 differential genes, comprising 432 upregulated and 417 downregulated genes (Figure 7A). Similarly, in the STZ versus maltol administration group, 1418 differential genes were identified, with 967 genes upregulated and 451 downregulated (Figure 7B). The red dots are upregulated differential genes, and the blue dots are downregulated differential genes, with *p*-values less than 0.05 (Figure 7A,B). The location of MME has been marked (Figure 7A,B). We focused on the common differential genes shared between these two groups for further investigation. Among these genes, we observed a significant differential expression of MME (Figure 7A,B). MME, also known as neprilysin, has been implicated in various pathological conditions, including diabetic nephropathy [23]. Given its differential expression and established role, MME emerges as a potential target for maltol in the context of diabetic complications.

### 2.8. Maltol Promoted the Expression of MME in HG/PA-Treated RSC96 Cells

**Figure 8:**
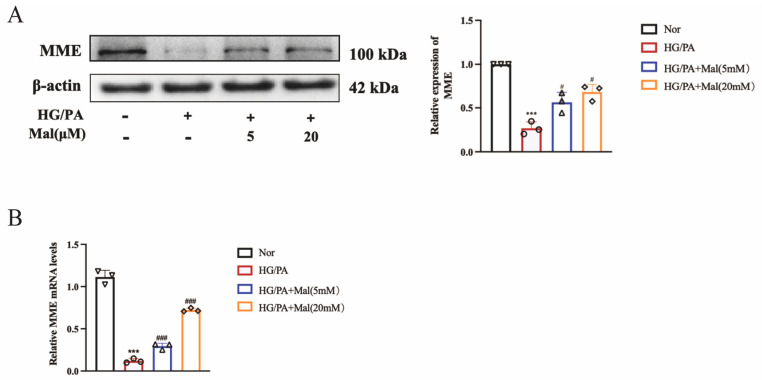

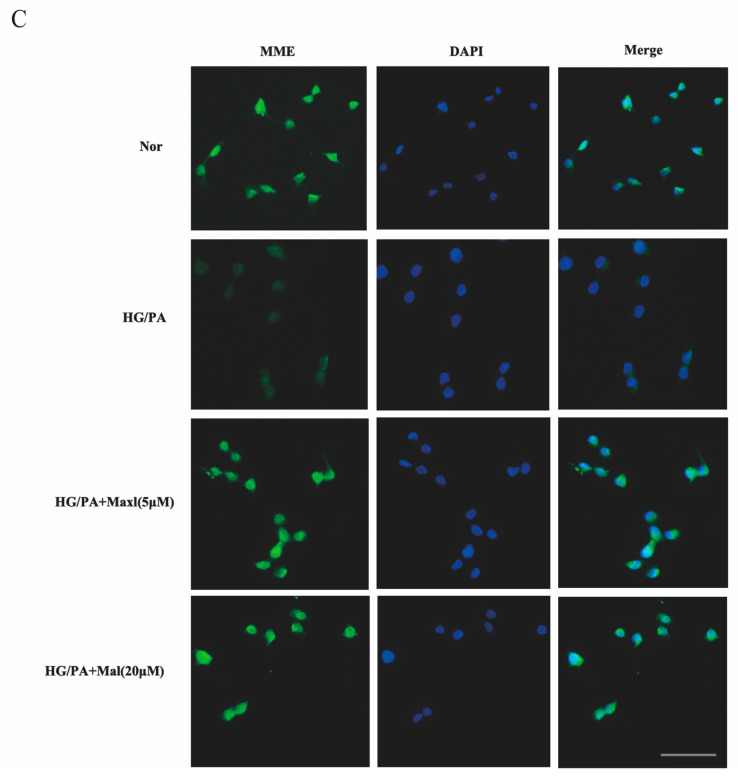
Maltol promoted the expression of MME in HG/PA-treated RSC96 cells. (**A**) Western blot detection of MME in RSC96 cells. (**B**) Real-time PCR detection of MME mRNA expression in RSC96 cells. (**C**) Immunofluorescence of MME expression in RSC96 cells. Scale bar = 100 μm. PA: palmitic acid; HG: high glucose; Mal: maltol. Symbols of each group represent the number of repetitions of the experiment. Data are expressed as mean ± SEM, *** *p* < 0.001 compared with Nor group; # *p* < 0.05, ### *p* < 0.001 compared with HG/PA group.

To investigate the effect of maltol on MME expression, MME mRNA expression in HG/PA-cultured RSC96 cells was assessed. The results indicated that HG and PA significantly reduced MME mRNA expression in RSC96 cells, whereas maltol treatment increased MME mRNA levels (Figure 8B). Additionally, MME protein expression in HG/PA-cultured RSC96 cells was evaluated using Western blot and immunofluorescence techniques (Figure 8A,C). The findings demonstrated that HG and PA downregulated MME protein expression in RSC96 cells (Figure 8A). Immunofluorescence further illustrated that maltol positively regulated MME protein expression in HG/PA-treated RSC96 cells (Figure 8C).

### 2.9. MME Downregulation Mediated Maltol-Inhibited Apoptotic Protein Expression in HG/PA-Treated RSC96 Cells

**Figure 9:**
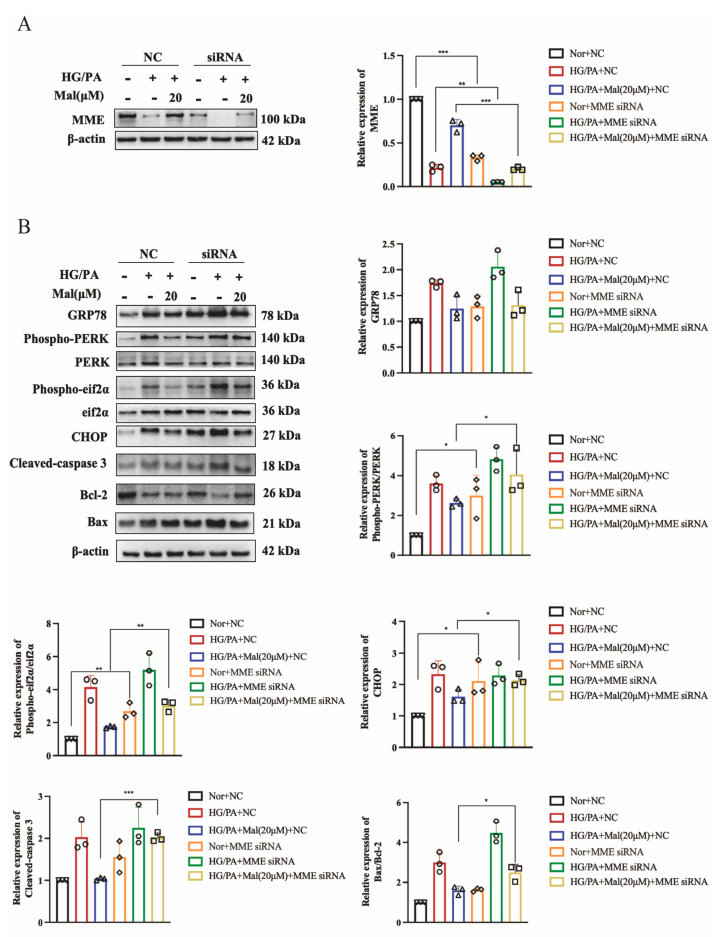

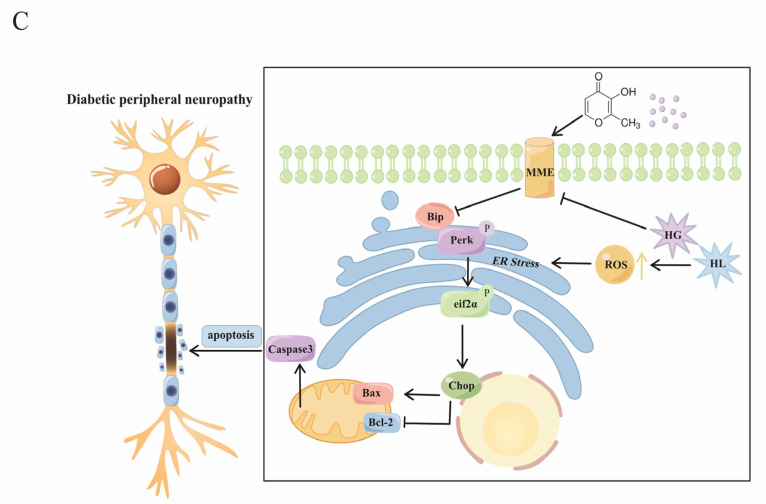
MME downregulation mediated maltol-inhibited apoptotic protein expression in HG/PA-treated RSC96 cells. (**A**) Western blot detection of MME in RSC96 cells transfected with blank siRNA and MME siRNA. (**B**) Western blot of GRP78, Phospho-eIF2α, eIF2α, Phospho-PERK, PERK, CHOP, Bax, Bcl-2, and Cleaved caspase-3 expression in RSC96 cells transfected with blank siRNA and MME siRNA. (**C**) Maltol’s efficacy in improving peripheral nerve function and reducing apoptosis in RSC96 cells in the context of DPN. PA: palmitic acid; HG: high glucose; Mal: maltol. Symbols of each group represent the number of repetitions of the experiment. Data are expressed as mean ± SEM, * *p* < 0.05, ** *p* < 0.01, *** *p* < 0.001 compared with HG/PA+Mal(20μM)+NC group.

To assess whether MME upregulation mediates maltol-induced anti-apoptotic effects, MME siRNA was utilized to transfect HG/PA-cultured RSC96 cells. Figure 9A shows that the transfection of MME siRNA significantly reduced MME protein expression in HG/PA-cultured RSC96 cells. Subsequently, the maltol-induced downregulation of Bax and Cleaved caspase-3 was enhanced by MME downregulation in HG/PA-cultured RSC96 cells, whereas Bcl-2 levels decreased (Figure 9A).

## 3. Discussion

Maltol is currently under extensive study for its implications in various diseases. As a food additive, maltol significantly mitigates organ toxicity induced by drugs. Specifically, maltol exhibits protective effects against cisplatin-induced cardiotoxicity by alleviating oxidative stress and reducing ROS-mediated apoptosis through the inhibition of the PI3K/Akt signaling pathway in cardiomyocytes [32]. Additionally, maltol has been observed to protect against hepatotoxicity via anti-inflammatory and anti-apoptotic signaling pathways [33]. Furthermore, in vitro and in vivo studies have demonstrated that maltol alleviates inflammatory conditions such as arthritis by inhibiting nuclear factor κB (NF-κB) signaling and activating nuclear factor-erythrocyte 2-associated factor-2 (Nrf2) [14]. In the models of aging induced by excessive ROS, maltol exhibits therapeutic effects by reducing the accumulation of advanced glycosylation end products (AGEs) in liver and kidney tissues, enhancing cell viability, and ameliorating aging-related cell cycle arrest caused by D-galactose [16]. Therefore, as an extract of red ginseng, maltol holds therapeutic promise in diseases characterized by oxidative stress and inflammation, with its primary pharmacological effects speculated to be anti-inflammatory and antioxidant.

Here, we demonstrate the neuroprotective effects of maltol on Schwann cells in DPN. Maltol improved nerve conduction velocity in diabetic mice. Previous studies by Samin Hong have reported that maltol mitigates oxidative damage in neuroretinal cells, with its neuroprotective and neurite outgrowth effects potentially linked to the NF-κB signaling pathway [18]. In conclusion, maltol presents itself as a promising new therapeutic agent for neuroprotection.

Additionally, we investigated maltol’s protective effects on Schwann cells, the key factor in myelin–axon coordination. Our findings indicate that maltol alleviates ER stress and apoptosis in HG/PA-cultured RSC96 cells. In the pathogenesis of DPN, hyperglycemia induces increased reactive oxygen species, triggering nitro-oxidative stress (NOS) and ER stress [34]. ROS can act as a signaling molecule involved in the process of autophagy, and excessive ROS levels can lead to lipid, protein and DNA damage [35]. Yin demonstrated that Astragaloside IV promotes autophagy and mitigates peripheral neuropathy by regulating the PI3K/Akt/mTOR signaling pathway through miR-155 [36]. Furthermore, Rapamycin, an immunosuppressive agent inducing autophagy, presents as a potential therapeutic avenue for DPN by affecting multiple cellular functions [37]. High blood-sugar-induced DNA damage can stimulate mitochondrial fission and aberrantly activate cell cycle components in post-mitochondrial neurons, leading to neuronal loss [38]. Hence, further investigation is warranted to ascertain whether maltol’s anti-apoptotic effects involve improvements in the mitochondrial function and regulation of autophagy in Schwann cells, thereby elucidating the precise mechanism underlying maltol’s beneficial effects in improving DPN.

Furthermore, we investigated maltol’s mechanism in enhancing ER stress-related genes GRP78 and CHOP in Schwann cells. Based on differential gene analysis using gene chip technology, MME emerged as a potential target of maltol in mitigating apoptosis and ER stress. The in vitro experiments confirmed that maltol reversed the downregulation of MME induced by high glucose in RSC96 cells. Additionally, the MME knockdown amplified maltol’s reduction of Bax and Cleaved caspase-3 expression in HG/PA-cultured RSC96 cells. MME has been implicated in modulating active signaling pathways via the PI3K/Akt pathway [39]. This study is the first to establish a connection between MME and diabetic peripheral neuropathy.

## 4. Materials and Methods

### 4.1. Materials

The primary antibodies used in this study were purchased from ProteinTech Group Inc (Rosemont, IL, USA): GRP78 (11587-1-AP), Phospho-EIF2S1 (28740-1-AP), EIF2S1 (11170-1-AP), Phospho-PERK (29546-1-AP), PERK (24390-1-AP), CHOP (15204-1-AP), BAX (50599-2-lg), Bcl2 (26593-1-AP), Cleaved Caspase 3 (25128-1-AP), and MME (18008-1-AP). The antibody against β-actin (BM3873) was sourced from Boster Biological Technology Co., Ltd. (Wuhan, China). Lipofectamine 2000 (11668019) was purchased from Thermo Fisher Scientific Inc (USA). Alexa Fluor 488-labeled Goat Anti-Rabbit IgG (H+L) (A0423) was acquired from Beyotime Biotechnology (Shanghai, China), and the anti-rabbit IgG secondary antibody (33201ES60) was obtained from Yeasen (Shanghai, China). Streptozotocin (STZ) was procured from Yeasen (Shanghai, China), while maltol (≥99.0%, W265608) was obtained from Sigma Chemical Co (St. Louis, MO, USA). Cell Counting Kit-8 (CCK-8) (C0043) was purchased from Beyotime Biotechnology (Shanghai, China). Hifair^®^ 1st Strand cDNA Synthesis SuperMix and Hieff UNICON^®^ qPCR SYBR Green Master Mix were sourced from Yeasen (Shanghai, China). MME siRNA was purchased from Gene Pharma Co., Ltd. (Shanghai, China). CCT020312 (HY-119240), a selective eIF2α/PERK activator, was obtained from MedChemExpress (Shanghai, China).

### 4.2. Animal Experiment

In the animal experiment, 50 male Sprague-Dawley (SD) rats weighing 150–170 g, obtained from Beijing HFK Bioscience Co. Ltd., Beijing, China, were housed under controlled conditions with a 12 h light/dark cycle and provided ad libitum access to food and water. After a 3-day acclimatization period, all rats were intraperitoneally injected with streptozotocin (STZ) at a dose of 65 mg/kg dissolved in citrate buffer (0.1 mol/L, pH 4.5; obtained from Yeasen, Shanghai, China) to induce hyperglycemia. Diabetes was confirmed by measuring blood glucose levels 3 days post-injection, with rats having random blood glucose levels >11.1 mmol/L classified as type I diabetic rats. The rats were then divided into five groups (*n* = 10 per group): normal rats treated with carboxymethyl cellulose and sodium (CMC-Na) (Nor), diabetic rats treated with CMC-Na (STZ), diabetic rats treated with maltol at doses of 25 mg/kg and 100 mg/kg (Mal 25 and Mal 100), and diabetic rats treated with α-lipoic acid (ALA) at a dose of 100 mg/kg (ALA 100), which served as the positive reference. Maltol and α-lipoic acid were administered orally once daily dissolved in 0.5% CMC-Na solution for a duration of 12 weeks, while normal and diabetic control groups received 0.5% CMC-Na solution alone. Throughout the treatment period, blood glucose levels and body weight were monitored dynamically to assess treatment efficacy and metabolic changes. The Experimental Animal Welfare Ethics Committee of the Institute of Materia Medica (Chinese Academy of Medical Sciences and Peking Union Medical College) under No. 00001042 approved all of the protocols for this research.

### 4.3. Measurement of MNCV

After 12 weeks of treatment, all rats were anesthetized with sodium pentobarbital (60 mg/kg, ip). The left sciatic nerve was exposed and stimulated at the proximal end using square-wave pulses (duration: 0.01 ms, intensity: 1 V) delivered through bipolar recording electrodes. Some rats died due to the procedure during the process, which could not be included in the statistical data. Action potentials at the distal end were recorded using the BL-420S biomechanical system (Chengdu Taimeng Technology Co., Ltd., Chengdu, China). Motor nerve conduction velocity (MNCV) was calculated using the formula:

MNCV (m/s) = (distance between stimulating and recording electrodes)/latency.

### 4.4. Measurement of the Thermal and Mechanical Hyperalgesia

After 12 weeks of treatment, thermal and mechanical hyperalgesia were assessed using the Hargreaves Apparatus 37370 and Dynamic Plantar Aesthesiometer 37450 (Ugo Basile S.R.L., Comerio, Italy). Rats were individually placed in six cages (17 × 11.5 × 14 cm), and their left hind paws were stimulated with infrared light (cut-off period set at 25 s) or a mechanical needle (biting strength set at 50 g). Each rat was tested five times with a 5 min interval between trials, and the mean value of withdrawal threshold or latency was recorded as the outcome measure. Some rats died due to the procedure during the process, which could not be included in the statistical data.

### 4.5. Measurement of Na^+^–K^+^-ATPase Activity

After 12 weeks of treatment, blood was taken from the heart to measure the sodium-potassium adenosine triphosphatase (Na^+^–K^+^-ATPase) activities in erythrocytes. For this assessment, 10 μL of whole blood was collected and mixed with 240 μL of distilled water. Na^+^–K^+^-ATPase activity was immediately determined using commercial kits purchased from Nanjing Jiancheng Bioengineering Institute (Nanjing, China). The determination and calculation of Na^+^–K^+^-ATPase activities were conducted according to the manufacturer’s instructions. Some rats failed to collect blood or died due to the procedure, which could not be included in the statistical data. 

### 4.6. Measurement of TAOC, MDA, GSH, and SOD

At the conclusion of the experiment, serum was isolated by centrifugation at 4 °C, 3000 rpm for 10 min. Subsequently, we determined the levels of total antioxidant capacity (TAOC), malondialdehyde (MDA), glutathione (GSH), and superoxide dismutase (SOD) in the serum, according to the manufacturer’s instructions. Total protein concentration in the cell lysates was determined using a bicinchoninic acid (BCA) protein assay kit (Beyotime, Shanghai, China) and used for the normalization of MDA levels. Some rats failed to collect blood or died due to the procedure, which could not be included in the statistical data.

### 4.7. Cell Culture and Groups

RSC96 cells, the rat Schwann cell line, were procured from the Cell Resource Center of the Shanghai Institutes for Biological Sciences, Chinese Academy of Sciences (Shanghai, China). The cells were cultured in Dulbecco’s modified Eagle’s medium (DMEM; MeilunBio, Dalian, China), supplemented with 10% fetal bovine serum (FBS; Gibco, Grand Island, NY, USA) and penicillin/streptomycin (100 units∙mL^−1^; Gibco), at 37 °C under a 5% carbon dioxide humidified atmosphere. RSC96 cells were divided into several experimental groups: normal glucose (5.5 mmol/L glucose), high glucose and high lipid (25 mmol/L glucose + 0.3 mM PA), high glucose and high lipid + maltol low-dose (25 mmol/L glucose + 0.3 mM PA, 5 μM maltol), high glucose and high lipid + maltol high-dose (25 mmol/L glucose + 0.3 mM PA, 20 μM maltol), and high glucose and high lipid + ALA (25 mmol/L glucose + 0.3 mM PA, 20 μM ALA). The effect of maltol on RSC96 cells was assessed, with relative detection performed 36 h after treatment. Additionally, to further elucidate the effect of maltol on ER stress, the PERK activator CCT020312 (HY-119240; MedChemExpress China) was employed. Furthermore, to clarify the role of MME in maltol-mediated inhibition of apoptosis, MME siRNA was transfected into RSC96 cells cultured under high glucose and PA conditions.

### 4.8. CCK-8 Assa

RSC96 cells were seeded into a 96-well plate at a density of 15,000 cells per well and cultured overnight in DMEM supplemented with 100 units/mL penicillin, 100 mg/mL streptomycin, and 10% (*v*/*v*) FBS. After treatment for 24h, 10 μL CCK-8 assay reagent was added to each well, and the cells were incubated for a further 2 h. Absorbance at 450 nm is then measured to determine cell viability, with the optical density (OD) value on the y-axis indicating cell viability.

### 4.9. Intracellular-ROS Generation Staining

Intracellular-ROS generation was assessed using the ROS Assay Kit (S0033, Beyotime, China) following the manufacturer’s protocol. After drug treatment, RSC96 cells were incubated with 10 μM DCFH-DA (1:1000 dilution) for 30 min in the dark at 37 °C, followed by three washes with PBS. Cells were then observed using a confocal microscope.

### 4.10. The Calcein-AM/PI Double Staining

The Calcein-AM /PI double staining kit (40747ES76, Yeasen, China) was used to distinguish between living and dead cells. RSC96 cells were incubated with 2 μM calcein-AM and 4.5 μM PI for 30 min. The cells were observed under a confocal microscope.

### 4.11. Cell Transfection

Cell transfection was performed according to Lipofectamine 2000 instructions. Specifically, 2 μL of siRNA was mixed with 100 μL Opti-MEM medium, and 5 μL Lipofectamine 2000 was mixed with 100 μL Opti-MEM medium. After an 18 min incubation at room temperature, the two solutions were combined and added to RSC96 cells. Indicated assays were conducted 72 h post-transfection.

### 4.12. Western Blotting

Total protein was extracted from RSC96 cells using RIPA lysis buffer. Protein content was assessed via immunoblotting. Proteins were separated by electrophoresis on 10% SDS-polyacrylamide gels and transferred onto polyvinylidene difluoride membranes. Membranes were blocked with 5% skim milk in 1× TBST for 1 h at RT, followed by overnight incubation at 4 °C with primary antibodies against GRP78 (1:5000), Phospho-EIF2S1 (1:5000), EIF2S1 (1:5000), Phospho-PERK (1:5000), PERK (1:5000), CHOP (1:1000), BAX (1:5000), Bcl-2 (1:1000), Cleaved Caspase 3 (1:1000), MME (1:1000), and β-actin (1:5000). After three washes with 1× TBST, membranes were incubated with horseradish peroxidase-conjugated secondary antibodies (anti-rabbit IgG, 1:10,000) for 1.5 h at RT. Following three additional washes with 1× TBST, protein bands were visualized and analyzed using Image Lab 6.1, with quantification normalized to β-actin levels. All experiments were performed independently at least three times.

### 4.13. Real-Time PCR

Total RNA was extracted from RSC cells using Trizol reagent (15,596, Invitrogen, Waltham, MA, USA) following the manufacturer’s protocol. The extracted RNA was reverse-transcribed to cDNA using Hifair^®^ II 1st Strand cDNA Synthesis SuperMix (Yeasen, Shanghai, China). PCR amplification was carried out using Hieff UNICON^®^ qPCR SYBR Green Master Mix (Yeasen, Shanghai, China). The specific primers are as follows: β-actin, 5′-GACCCAGATCATGTTTGAGACC-3′ (forward) and 5′-AGGCATACAGGGACAACACA-3′ (reverse); CHOP, 5′-CTGAAGAGAACGAGCGGCTCAAG-3′ (forward) and 5′-GACAGGAGGTGATGCCAACAGTTC-3′ (reverse); GRP78, 5′-ACACCTGACCGACCGCTGAG-3′ (forward) and 5′-GCCAACCACCGTGCCTACATC-3′ (reverse); Mme, 5′-GCCAAGCATACAGAGCCTATCAG-3′ (forward) and 5′-ACACCTGGGCAAAGTTCAAGAAG-3′ (reverse); Ngf, 5′-CTGGACCCAAGCTCACCTCA-3′ (forward) and 5′-GTGGATGAGCGCTTGCTCCT-3′ (reverse); Nrnl, 5′-GGGCGAAAGATATGTGGGAT-3′ (forward) and 5′-CGAGAGAGACACCAGGAGCA-3′ (reverse); NSE, 5′-GAACTATCCTGTGGTCTCC-3′ (forward) and 5′-CGACATTGGCTGTGAACTTG-3′ (reverse).

### 4.14. Immunofluorescence

RSC96 cells were cultured on cover glasses in coverglass-bottom dishes. Following treatment, cells were fixed with 4% paraformaldehyde at room temperature for 15 min. Permeabilization was achieved by incubating cells with 0.3% Triton X-100 (Beyotime, Shanghai, China) in PBS for 10 min at room temperature. Subsequently, cells were incubated overnight at 4 °C with primary antibodies. After washing with PBS, cells were incubated with Alexa Fluor 488-labeled Goat Anti-Rabbit IgG (H+L) secondary antibodies for 2 h at 37 °C. Following counterstaining with 4′,6-diamidino-2-phenylindole (DAPI), RSC96 cells were observed using an inverted fluorescence microscope.

### 4.15. Gene Chip

Sciatic nerve tissue from rats was analyzed using a gene chip. Total RNA was quantified using NanoDrop ND-2000 (Thermo Scientific, Waltham, MA, USA), and RNA integrity was assessed via agarose gel electrophoresis. For microarray analysis, total RNAs were transcribed to double-stranded cDNAs, followed by the synthesis of cRNAs. Subsequently, second-cycle cDNAs were synthesized from cRNAs. After fragmentation and biotin labeling, the second-cycle cDNAs were hybridized onto the microarray. Arrays were scanned using the Affymetrix Scanner 3000 (Affymetrix 7G), and data analysis was performed using Command Console software (version 4.0, Affymetrix, Santa Clara, CA, USA) for raw data extraction, followed by RMA normalization using Expression Console software (version 1.4.1, Affymetrix). Differential gene expression analysis was conducted based on fold change and *p*-value calculated using *t*-tests (threshold *p*-value < 0.05). Volcano plots were generated to illustrate upregulated and downregulated DEGs (adj. *p*. Value < 0.05, LogFC > 1 or <−1). GO and KEGG analyses were performed to determine the biological roles of identified genes.

### 4.16. Statistical Analyses

Data involving more than two groups were analyzed using one-way ANOVA, while differences between two groups were assessed using Student’s *t*-test. All statistical analyses were conducted using GraphPad Prism (ver. 5.0; GraphPad Software, San Diego, CA, USA) with data derived from at least three independent samples. A *p*-value < 0.05 was considered statistically significant.

## 5. Conclusions

Our findings represent the inaugural demonstration of maltol’s efficacy in improving peripheral nerve function and reducing apoptosis in RSC96 cells in the context of DPN. The results indicate that maltol’s anti-apoptotic effect involves the modulation of ER stress. Moreover, MME, upregulated by maltol, mediates the expression of ER stress and apoptosis-related proteins inhibited by maltol (Figure 9B). In conclusion, maltol emerges as a promising therapeutic agent for safeguarding against Schwann cell dysfunction and managing diabetic peripheral neuropathy.

## Data Availability

The original contributions presented in the study are included in the article, further inquiries can be directed to the corresponding authors.
